# “Role of the adipocyte hormone leptin in cardiovascular diseases – a study from Chennai based Population”

**DOI:** 10.1186/s12959-015-0042-4

**Published:** 2015-03-04

**Authors:** Devarajan Nalini, Rajendran Karthick, Vijay Shirin, Ganesan Manohar, Raghunathan Malathi

**Affiliations:** Department of Genetics, Dr. ALM PG Institute of Basic Medical Sciences, University of Madras, Taramani, Chennai, 600 113 India; Department of Cardiology, Stanley Medical College and Hospital, Chennai, 600 001 India

**Keywords:** Leptin, Insulin, Tumor necrosis factor-α, Obesity, Acute Myocardial Infarction, Cardiovascular Diseases

## Abstract

**Background:**

Obesity is currently regarded as a pro-inflammatory condition during which leptin (*Ob* gene product) might act as a risk factor for Cardiovascular Diseases (CVD) including Acute Myocardial Infarction (AMI). There is a marked increase in circulating leptin concentrations and inflammatory markers such as Tumor Necrosis Factor-α (TNF-α) in AMI patients but still the association of leptin with inflammation during AMI is not known. The present study suggest that elevated levels of leptin might elicit the risk for CVD by signaling for the secretion of inflammatory cytokines especially, TNF-α.

**Methods:**

Blood samples were collected from 100 CVD subjects diagnosed for AMI immediately after their admission to the hospital and serum leptin, insulin, glucose, lipids and inflammatory marker such as TNF-α were measured. 5 ml random (non-fasting) blood was collected from 100 non-CVD (control) subjects and the results obtained in case of AMI subjects were compared with that of the control subjects. The subjects under study included both men and women belonging to the age group of 35 – 75 and they were classified based on their BMI as normal weight, overweight and obese.

**Results:**

Circulating levels of leptin are found to be elevated in obese control subjects and in patients with AMI irrespective of their Body Mass Index (BMI). In addition, leptin is also found to be positively correlated to serum triglycerides, insulin and TNF-α in AMI subjects. MANOVA analysis suggests that leptin might influence the synthesis of insulin and TNF-α. This is the first report relating leptin to TNF-α in Chennai based population, India.

**Conclusions:**

Hyperleptinemia might act as a risk marker for AMI. The present study suggests that at elevated levels, leptin may favor atherosclerosis by promoting the synthesis of TNF-α and insulin. However, our report warrants further investigation both *in vitro* and *in vivo* to determine the exact mechanism behind the pro-atherogenic role of leptin. The observed positive correlation between leptin and BMI in both AMI and control subjects suggests that obese subjects manifest leptin resistance and hence, they possess a greater risk for the incidence of CVD.

## Background

Obesity, an emerging epidemic of industrialized countries is currently proclaimed to be a pro-inflammatory state with inflammation playing an active role in the pathophysiology of Cardiovascular Diseases (CVD) [1,2]. But still, the key mechanisms that link elevated fat mass with inflammation during CVD including AMI remain elusive. Leptin, a 16 KDa protein hormone is a novel and very promising molecule of research that may act as a mediator between obesity and CVD [3]. It is one of the most important adipocytokine that modulates metabolic processes by regulating energy intake and expenditure, including appetite and metabolism [4]. Apart from metabolism, leptin has systemic effects including regulation of angiogenesis, wound healing, lipolysis, blood pressure homeostasis, reproduction, hematopoiesis and immune function [5]. Despite the weight reducing effects of leptin, obese individuals possess unusually high concentrations of circulating leptin which in turn is indicative of leptin resistance.

Leptin signaling pathway is a complex network that regulates a cellular pathway involved in a myriad of physiological and pathological scenarios. Leptin transmits signal by binding to its receptor Ob-R, which belongs to the class I cytokine receptor family. The long form, namely Ob-Rb is essential in mediating most of the biological effects of leptin and is highly expressed in the hypothalamus. It is also found to be expressed in several cell types relevant to CVD (eg, macrophage, endothelial cell, and smooth muscle cells) thus providing evidence for the role of leptin in signaling for atherogenic events [6].

Serum leptin levels are reported to be associated with various cardiovascular risks, including stroke, chronic heart failure [7,8], acute myocardial infarction [9], coronary heart disease [10], and left cardiac hypertrophy [11]. It is an important factor operating in the metabolic alteration taking place during myocardial infarction and is a possible risk factor [12-14]. One of the molecular mechanism by which leptin promotes the onset of AMI is its ability to modulate immune response. Evidence suggests that leptin can stimulate inflammatory response by activating TNF-α via p38 and JNK MAPK pathway [15] and these inflammatory markers may be associated with the risk of recurrent myocardial infarction and death [16]. The normal physiological effect of leptin on the regulation of TNF-α expression seems to be suppressive, but the hyperleptinemic condition and leptin resistance may both contribute to the rise of TNF-α in the adipose tissue in obesity [17]. Also, several pieces of evidence indicate that TNF-α is an important player in the state of insulin resistance observed during obesity which in turn contributes to several pathological problems of obese patients such as hyperlipidemia, arteriosclerosis and hypertension [18,19]. Given that both of these cytokines are overproduced in the adipose tissue of obese individuals and released into the circulation, they may augment inflammatory response and could be involved in the pathogenesis of the CVD during obesity.

Epidemiological studies show striking differences in the extent and severity of CVD when people from different populations are compared. The present study examines the relationship between obesity, leptin and AMI in Chennai based population. The present study focuses on the following 1. To determine the values of leptin, insulin and TNF-α in AMI and control subjects of different BMI 2. To understand the pro-atherogenic role of leptin by analyzing its influence over the secretion of insulin, TNF-α and triglycerides.

## Methods

The relationship between leptin, insulin, biochemical and inflammatory markers were studied in subjects with AMI (CVD subjects) and control (non-CVD) subjects. About 100 CVD patients considered for the present study were diagnosed for AMI and admitted to ICCU, Stanley Medical College and Hospital, Chennai. The study population included 79 men and 21 women belonging to the age group of 35 – 75 and AMI was diagnosed in these patients based on acute changes in ST elevations in ECG. Detailed history was recorded from the patients with reference to the features of chest pain, location, radiation, aggravating and relieving factors, increased autonomic activity and other clinical features. The diagnosis of AMI was based on clinical and electrographic evidence using the criteria recommended by WHO. Trained nurses administered questionnaires to obtain information on each patient’s date of birth, occupation, current cigarette smoking and alcohol use. After obtaining a written consent, random blood samples (5 mL) were collected from the AMI subjects at the time of their admission to ICCU, Stanley Medical College and Hospital, Chennai. Blood was then allowed to clot and it was retracted and separated by centrifugation at 3500 rpm for 10 minutes. Serum was separated using standard protocol and stored at −80°C.

100 control (non-CVD) subjects chosen for the present study were those who had no history of incidence of CVD. The study population consisted of 77 men and 23 women belonging to the age group of 35 – 75 and was devoid of diabetes, any heart disease, thyroid disorders and arthritis. None of the subjects were on medication known to affect insulin action or plasma lipoprotein – lipid levels and they were not on any inflammatory drug either before or at the time of the study. People with the habits of smoking and alcohol consumption and the individuals using aspirin as a chronic medication were excluded from the study and the subjects were under no medication for at least 3 months before the study. Written consent was obtained from the above subjects to participate in the present study. Trained interviewers administered questionnaires to obtain information on each subject’s date of birth, occupation, current cigarette smoking and alcohol use. 5 ml of random (non-fasting) blood samples were collected from them, serum was separated using standard protocols and stored at −80°C.

All the subjects under study were categorized based on BMI, as normal weight, overweight and obese. The cut off range for BMI is as follows: normal weight - 18.5-22.99; overweight - 23.0-27.49; obese - >27.5.

BMI was calculated using the formula – Weight (Kg)/Height (m^2^). Waist to Hip ratio (WHR) was calculated as waist circumference (in inches) divided by inch circumference (in inches). Blood pressure was measured using mercury sphygmomanometer.

### Biochemical parameters

Serum parameters such as glucose was estimated using GOD-POD method [20], triglycerides using GPO-POD-ESPT method [21], cholesterol using CHOD-POD method [22], HDL-cholesterol using glycerol-3-phosphate oxidase – peroxidase-N-ethyl-methylanilin propan-sulphonate sodic method using auto analyzer (BAYER RA 50; Bayer Company India, Guindy, Chennai, India).

### ELISA method

Serum leptin (Diagnostics Biochem Canada Inc. Canada), insulin (Cal biotech, California) and TNF-α (Orgenium Laboratories Business Unit, Finland) were measured using Sandwich ELISA technique according to the manufacturer’s instructions.

### Statistical analysis

Data obtained are presented as mean ± SD. As the data obtained was not normally distributed, Mann–Whitney U test was used to assess the difference in values between AMI and control subjects. The relationship between continuous variables was evaluated using Spearman’s rank correlation technique. The role of leptin on the incidence of CVD was evaluated by analyzing its influence over established cardiovascular risk factors such as insulin, TNF-α and triglycerides using Multivariate Analysis of Variance (MANOVA). A value of p < 0.05 is considered statistically significant.

## Results

Table 1 shows the list of values of BMI, serum leptin, insulin, TNF-α, glucose and triglycerides in AMI and control subjects. In the present study, values of serum leptin are found to be two to three fold higher in AMI subjects when compared to that of control (non-CVD) subjects. Also, a strong positive correlation is observed between serum leptin and BMI in both AMI (r = 0.57, p < 0.0001; Table 2) and control subjects (r = 0.54, p < 0.0001). Interestingly, a significant difference (p < 0.0001) in serum leptin levels was observed between men (22.3 ± 6.2 ng/ml) and women (37.4 ± 7.3 ng/ml) in case of AMI group. However, no such difference was observed between men (16.1 ± 5.3 ng/ml) and women (15.0 ± 5.2 ng/ml) of control group.

**Table 1:** **Values of serum leptin, insulin, TNF-α, glucose, triglycerides, cholesterol, HDL, LDL and VLDL in AMI and control (non-CVD) subjects**

**BMI CLASS (Kg/m** ^**2**^ **)**		**LEPTIN (ng/mL)**	**INSULIN (μIU/mL)**	**TNF-α (pg/mL)**	**GLUCOSE (mg/dL)**	**TRIGLYCERIDES (mg/dL)**	**CHOLESTEROL (mg/dL)**	**HDL (mg/dL)**	**LDL (mg/dL)**	**VLDL (mg/dL)**
Normal Weight	Control Subjects (21)	5.7 ± 3.8	2.6 ± 0.97	14.6 ± 2.3	124.6 ± 14.3	190.4 ± 26.8	37.8 ± 7.95	122.75 ± 10.2	26.5 ± 11.1	122.75 ± 10.2
	AMI Subjects (58)	19.4 ± 3.3	13.4 ± 3.3	37.9 ± 5.2	152.3 ± 18.6	200.9 ± 19.7	40.54 ± 9.2	129.8 ± 8.6	28.6 ± 13	129.8 ± 8.6
	p Value^#^	<0.0001****	<0.0001****	<0.0001****	0.3415	0.37	0.415	0.29	0.8	0.29
Over Weight	Control Subjects (46)	10.5 ± 4.2	3.4 ± 1.8	14.2 ± 2.1	144.13 ± 21.3	174.4 ± 23.4	35.1 ± 9.8	112.02 ± 12.4	28.8 ± 13.3	112.02 ± 12.4
	AMI Subjects (29)	31.7 ± 5.2	15 ± 5.8	41.8 ± 6.5	186.3 ± 19.6	221.65 ± 27.8	43.9 ± 9.3	134.6 ± 15.2	38.2 ± 14.9	134.6 ± 15.2
	p Value^#^	0.0007***	<0.0001****	<0.0001****	0.004**	0.0008***	0.0009***	0.008**	0.03*	0.008**
OBESE	Control Subjects (33)	26.9 ± 9.1	7.1 ± 4.4	15.95 ± 1.9	167.9 ± 25.8	193.4 ± 23.4	37.97 ± 10.22	117.2 ± 17.2	32.5 ± 16.5	117.2 ± 17.2
	AMI Subjects (13)	51.5 ± 12.4	31.35 ± 7.5	46.8 ± 5.9	219.4 ± 21.4	221 ± 28.32	39.7 ± 10.3	135.9 ± 12.6	33 ± 10.9	135.9 ± 12.6
	p Value^#^	0.02*	<0.0001****	<0.0001****	0.003**	0.18	0.95	0.03*	0.52	0.03*

*p<0.05; **p<0.01; ***p<0.001; ****p<0.0001.

**Table 2:** **Correlation of serum leptin, insulin, TNF-α, glucose and triglycerides with BMI in AMI and control subjects**

	**LEPTIN**		**INSULIN**		**TNF-α**		**GLUCOSE**		**TRIGLYCERIDES**	
	**AMI Subjects**	**Control subjects**	**AMI Subjects**	**Control subjects**	**AMI Subjects**	**Control subjects**	**AMI Subjects**	**Control subjects**	**AMI Subjects**	**Control subjects**
**BMI**	0.57****	0.54****	0.32**	0.39****	0.24*	0.13	0.24*	0.12	0.5****	0.2*

*p<0.05; **p<0.01;****p<0.0001.

Serum insulin levels (Table 1) are found to be highly elevated in AMI subjects when compared to that of control subjects. A notable finding is that serum insulin levels are within the normal range in control whereas in AMI subjects, its level is significantly higher. Serum insulin is found to exhibit a good positive correlation with BMI (Table 2) in both AMI (r = 0.32, p = 0.001) and control subjects (r = 0.39, p < 0.0001). Table 3 shows that there is also a positive correlation between serum insulin and leptin in both AMI (r = 0.27, p = 0.006) and control subjects (r = 0.22, p = 0.03).

**Table 3:** **Correlation of serum insulin, TNF-α and triglycerides with leptin in AMI and control subjects**

	**INSULIN**		**TNF-α**		**TRIGLYCERIDES**	
	**AMI Subjects**	**Control subjects**	**AMI Subjects**	**Control subjects**	**AMI Subjects**	**Control subjects**
**LEPTIN**	0.27**	0.22*	0.27**	0.15	0.24*	0.03

*p<0.05; **p<0.01.

Serum TNF-α level was measured in AMI subjects and the results obtained were compared with that of control (non-CVD) subjects (Table 1). Its values are found to be three fold higher in AMI when compared to control subjects. Tables 2, 3 and 4 show that serum TNF-α exhibits a direct correlation with BMI (r = 0.24, p = 0.02), leptin (r = 0.27, p = 0.007) and insulin (r = 0.21, p = 0.03) respectively in AMI subjects but such a correlation was not observed in case of control subjects.

**Table 4:** **Correlation of TNF-α with insulin in AMI and control subjects**

	**TNF-α**	
	**AMI Subjects**	**Control subjects**
**INSULIN**	0.21*	0.24*

*p<0.05.

Insulin, TNF-α and triglycerides are implicated in the pathophysiological processes of CVD. To elucidate the possible mechanism by which leptin promotes the onset of AMI, its influence over the secretion of insulin, TNF-α and triglycerides were analyzed using MANOVA in AMI subjects and control subjects. MANOVA revealed a significant main effect for leptin, (Wilks λ = 0.931, F (3, 200.00) = 4.975, p = 0.002, partial eta squared = 0.069) in AMI subjects. Power to detect the effect was 0.910. Given the significance of the overall test, the univariate main effects of leptin were examined. Significant univariate main effects for leptin was obtained for insulin (F = 9.8, p = 0.002, partial eta square = 0.046, power = 0.876) and TNF-α (F = 3.92, p =0.05, partial eta square = 0.019, power = 0.505) in AMI subjects. Thus, MANOVA analysis show that leptin influence the secretion of insulin (p = 0.002) and TNF-α (p = 0.05) in AMI subjects.

Analysis of biochemical parameters has yielded some interesting results. Serum glucose levels (Table 1) are found to be higher in AMI when compared to that of control subjects of different BMI. Overweight and obese AMI subjects are also found to possess a higher value of serum triglycerides when compared to control subjects. However, there is no significant difference in serum triglycerides between normal weight control and AMI subjects. Also interestingly, a positive correlation is observed between serum triglycerides and leptin in AMI subjects (r = 0.24, p = 0.03) (Table 3) but not in control subjects.

Serum values of cholesterol, HDL and VLDL are found to be higher in overweight AMI when compared to control subjects. Serum LDL level is found to be in normal range in case of control subjects irrespective of their BMI but its value is significantly higher in overweight AMI and obese AMI subjects.

Table 5 illustrates the values of systolic and diastolic blood pressure in AMI and control subjects. Both systolic and diastolic blood pressure are found to be higher in AMI subjects than in control subjects.

**Table 5:** **Values of systolic and diastolic blood pressure, waist to hip ratio in AMI and control subjects**

**BMI CLASS (Kg/m** ^**2**^ **)**	**SYSTOLIC BP (mm Hg)**	**DIASTOLIC BP (mm Hg)**	**WAIST TO HIP RATIO**
AMI subjects (100)	139.4 ± 25.7	93.3 ± 15.4	0.94 ± 0.06
Control subjects (100)	125.4 ± 20.04	85.8 ± 14.8	1.01 ± 0.09
P value^#^	0.0001***	0.0003***	0.0001***

****p<0.001.

Waist to hip ratio was also measured and interestingly it is found to exhibit a significant positive correlation with triglycerides (r = 0.26, p = 0.009) and VLDL (r = 0.35, p = 0.0001) only in the case AMI subjects. No such correlation is observed in the case of control subjects (Table 6).

**Table 6:** **Correlation of serum triglycerides and VLDL with waist to hip ratio in AMI and control subjects**

	**TRIGLYCERIDES**		**VLDL**	
	**AMI Subjects**	**Control subjects**	**AMI Subjects**	**Control subjects**
**WAIST TO HIP RATIO**	0.26**	0.02***	0.35	0.01

**p<0.01; ***p<0.001.

## Discussion

Obesity is a leading cause of preventable death, a growing epidemic and major contributor to CVD risk and mortality in developed and developing nations [23]. It is associated with a marked increase in circulating leptin concentrations (*Ob* gene product) but the pathophysiological mechanisms linking obesity and CVD are poorly defined. Leptin receptors are expressed in atherosclerotic lesions, and leptin signaling is implicated in the promotion of both thrombosis and atherosclerosis in experimental models, suggesting a role for leptin in the progression of CVD [24]. In the present study, we analyzed the serum levels of leptin and its influence over insulin, TNF-α and triglycerides in CVD patients and control subjects in order to understand the role played by leptin in the pathogenesis of CVD.

Recent evidences indicate that under normal conditions leptin may be an important factor in regulating energy balance [25], but during situations of hyperleptinemia, this hormone may function pathophysiologically and might lead to progression of CVD [26]. This may be mediated through various atherogenic effects of leptin including its effect on blood pressure [27], platelet aggregation [28], formation of arterial thrombosis [29] and inflammatory vascular response [30]. Also, high levels of leptin are believed to be associated with lower arterial distensibility, an index of circulatory function and are found to be involved in the pathogenesis of atherosclerotic process [31]. In the present study, serum leptin is found to be two to three fold higher in AMI subjects when compared to control subjects in all the categories of BMI (viz., normal weight, overweight and obese). These findings suggest that individuals with elevated leptin levels may have a greater risk for the incidence of CVD. Also interestingly, serum leptin levels were found to be significantly higher in women with AMI than in men with AMI. The mechanism behind this gender differences is not fully understood, but still, the sex hormones may be the most probable candidates in this regard [32].

Leptin is also found to be positively correlated to BMI in both AMI and control subjects. The observed elevation in leptin levels during obese conditions suggests that they are leptin resistant and hence, they possess a greater risk for the incidence of CVD. Another interesting observation is that serum leptin level is higher in normal weight AMI subjects than in overweight control subjects. This prominent rise in serum leptin levels suggest that metabolic parameters other than obesity might influence leptin secretion during pathological conditions thereby resulting in leptin resistance.

CVD is demonstrated to be characterized by resistance to insulin mediated glucose disposal. Insulin resistance as well as compensatory hyperinsulinemia associated with insulin resistance is shown to be independent predictors for CVD [33]. In the present study, serum insulin values are found to be higher in AMI subjects than in control subjects. Interestingly, serum glucose is also found to be high in them in spite of higher insulin levels. Thus, elevated levels of glucose along with increase in insulin suggest that metabolic conditions such as hyperinsulinemia could be an added risk for the incidence of CVD. Moreover, serum insulin is found to exhibit a significant positive correlation with BMI and leptin in both AMI and control subjects thereby suggesting that hyperleptinemia might be associated with the development of obesity and subsequent metabolic abnormalities such as hyperinsulinemia.

TNF-α is one of the most potent pro-inflammatory cytokine that play a pathogenic role in chronic inflammatory diseases [34]. It is reported to be involved in every step of inflammation from initiation to downregulation and each characteristic lesion of atherosclerosis [35]. In the present study, serum TNF-α level is found to be threefold higher AMI subjects than in control subjects. It is also found to be positively correlated to leptin in AMI subjects thereby suggesting that leptin might cause an increase in TNF-α expression which in turn might exert a pro-atherogenic role in CVD.

To elucidate the possible mechanism by which leptin could promote the onset of CVD, its influence over the secretion of insulin, TNF-α and triglycerides were analyzed using MANOVA in CVD subjects and control subjects. MANOVA analysis suggests that leptin might exert its pro-atherogenic role by stimulating the secretion of TNF-α and insulin. Our reports may be regarded as preliminary data and an in-depth study both *in vivo* and *in vitro* will enable us to broaden our vision over the pro-inflammatory role of leptin during the pathogenesis of CVD.

In the present study, serum triglycerides are found to be higher in AMI subjects than in control subjects. In addition, serum leptin levels are found to be positively correlated to serum triglycerides in AMI subjects. Moreover, serum cholesterol, HDL, LDL and VLDL are found to be higher in overweight AMI subjects when compared to that of control subjects. Higher VLDL triglyceride output are reported to activate cholesteryl ester transfer protein, which in turn result in triglyceride enrichment of LDL and HDL [36] and the small, dense LDL particles are demonstrated to be more susceptible to oxidative modification, [37,38]. In addition, waist to hip ratio is also found to exhibit a significant positive correlation to triglycerides and VLDL in AMI subjects but such a correlation is not observed in control (non-CVD) subjects. All these observations suggest that increased visceral obesity in association with elevated serum levels of leptin, insulin, TNF-α, triglycerides, and VLDL may act synergistically to increase one’s risk of acquiring CVD.

Systolic and diastolic blood pressure are found to be higher in AMI subjects than in control subjects. Evidences suggest that uncontrolled and prolonged elevation of blood pressure in AMI can lead to a variety of changes in the myocardial structure, coronary vasculature, and conduction system of the heart (http://emedicine.medscape.com/article/162449-overview). These changes in turn can lead to the development of LVH, CAD, various conduction system diseases, and systolic and diastolic dysfunction of the myocardium, complications that manifest clinically as angina or myocardial infarction.

Taken together, in the present study, circulating levels of leptin are found to be associated with well defined cardiovascular risk factors such as obesity, triglycerides, insulin and TNF-α in AMI subjects. Our results suggest that leptin may act as a risk factor for CVD and might mediate obesity and CVD by augmenting inflammatory events. However, further studies focusing on the pro-inflammatory role of leptin during the pathogenesis of AMI will aid in the development of leptin based therapeutic strategies to combat CVD.

## Abbreviations

CVD: Cardiovascular disease
AMI: Acute myocardial infarction
TNF-α: Tumor necrosis factor – alpha
BMI: Body mass index
BP: Blood pressure
MANOVA: Multivariate analysis of variance.
